# Influence of quartz powder and silica fume on the performance of Portland cement

**DOI:** 10.1038/s41598-020-78567-w

**Published:** 2020-12-08

**Authors:** Ludmila Rodrigues Costa Tavares, Joaquim Francisco Tavares Junior, Leonardo Martins Costa, Augusto Cesar da Silva Bezerra, Paulo Roberto Cetlin, Maria Teresa Paulino Aguilar

**Affiliations:** 1 Universidade Federal de Minas Gerais, Av. Antônio Carlos, 6627 - Pampulha, Belo Horizonte, Minas Gerais CEP 31 270-901 Brasil; 2 Centro Federal de Educação Tecnológica de Minas Gerais, Av. Amazonas, 5253 – Nova Suíça, Belo Horizonte, Minas Gerais CEP 30421-169 Brasil

**Keywords:** Civil engineering, Mechanical properties

## Abstract

Supplementary cementitious materials interact chemically and physically with cement, influencing the formation of hydrate compounds. Many authors have analyzed the filler and pozzolanic effect. However, few studies have explored the influence of these effects on hydration, properties in the fresh and hardened states, and durability parameters of cementitious composites separately. This study investigates the influence of the replacement of 20% of Portland cement for silica fume (SF) or a 20-µm medium diameter quartz powder (QP) on the properties of cementitious composites from the first hours of hydration to a few months of curing. The results indicate that SF is pozzolanic and that QP has no pozzolanic activity. The use of SF and QP reduces the released energy at early times to the control paste, indicating that these materials reduce the heat of hydration. The microstructure with fewer pores of SF compounds indicates that the pozzolanic reaction reduced pore size and binding capability, resulting in equivalent mechanical properties, reduced permeability and increased electrical resistance of the composites. SF and QP increase the carbonation depth of the composites. SF and QP composites are efficient in the inhibition of the alkali-aggregate reaction. The results indicate that, unlike the filler effect, the occurrence of pozzolanic reaction strongly influences electrical resistance, reducing the risk of corrosion of the reinforcement inserted in the concrete.

## Introduction

Cementitious composites with a low environmental impact associated with high mechanical performance and good durability indicators can be produced using supplementary cementitious materials (SCM)^1–6^. In these composites, SCM can act as cementing, pozzolanic or filler materials^7,8^. SCM with cementing properties, such as blast furnace slag, result in hydrous compounds with similar chemical composition and structure to Portland cement hydration products^9^. Pozzolanic materials, such as silica fume, react chemically with the calcium hydroxide generated in cement hydration and produce additional calcium silicate hydrate (C–S–H) and other hydrated compounds (pozzolanic reaction)^7,10,11^. This additional C–S–H fills the gaps in the paste by promoting pore refinement and cementitious matrix thickening^12^.

Non-pozzolanic high-fineness inorganic materials (such as limestone and finely ground quartz), and even pozzolans, cause the so-called filler effect, that is, they fill voids in the cement paste microstructure and physically stimulate cement hydration in the first few hours of curing, densifying the structure of the cementitious matrix^8,13–18^. These materials act as nucleation and growth point of C–S–H and other hydrates^19,20^. Another mechanism associated with the filler effect is the dilution of clinker in the cement paste, which provides extra space for compounds formed in the hydration of the clinker^8,21,22^. Limestone filler, although not pozzolanic, reacts chemically in hydrated cement, forming secondary hydrated compounds and influencing the formation of C–S–H^13^. Thus, to isolate the influence of the filler effect and pozzolanic activity on the different properties of a Portland cement composite is complex. This understanding could be important for dosing concretes and mortars, aiming a better mechanical performance and durability. Using thermal analysis by calorimetry and thermogravimetry and their derivatives, different authors have shown that the filler effect of SCM is more pronounced than pozzolanic activity in the first hours of hydration, while pozzolanic activity predominates after one day of curing^19,23,24^. However, studies still diverge on the effects of using SCM with and without pozzolanic activity on concrete durability indicators, such as electrical resistance^25^ and accelerated carbonation depth^9,26,27^.

Finely ground quartz particles may have a pozzolanic activity or may only provide a filler effect depending on their particle size and specific surface. However, there is no consensus in the literature on the critical size required for quartz to have a pozzolanic activity^28^. Benezet and Benhassaine^29^, utilizing the Chapelle test, concluded that quartz particles with a diameter d_50_ below 5 µm are pozzolanic. Berodier and Scrivener^13^ indicated, by isothermal calorimetry and scanning electron microscopy (SEM), that quartz samples with diameters d_50_ of 4, 13 and 18 µm replacing 20–70% of cement may physically stimulate clinker hydration in the first hours by the filler effect. Kadri et al.^30^, in turn, indicated that 10% of cement replacement by quartzite (~ 75% quartz) with a diameter d_50_ of 2.6, 5.5 and 11 µm had little impact on semi-adiabatic calorimetry observed. Thus, quartz particles with a diameter between 20 µm and 25 µm are expected to have no pozzolanic activity, but to act as a filler material. The use of raw material as a filler can reduce energy consumption in the cement industry, leading to a more sustainable process^31^. In this context, this study evaluates the performance of cementitious composites with and without partial replacement of cement by finely ground quartz with negligible pozzolanic activity and a high pozzolanic activity material (silica fume) from the first hours of hydration to 300 days of curing. Thus, the mechanisms that strongly influence the performance of these composites are identified.

## Materials and methods

### Materials

The materials used in this study were Portland cement (PC), silica fume (SF), quartz powder (QP), and fine aggregate. The SF was considered for comparison with the QP due to the similarity in the chemical composition. Other pozzolans were considered to carry out this work, such as metakaolin, fly ash and sugar cane bagasse ash. However, these pozzolans were disregarded due to the significant differences in chemical composition compared to quartz powder, mainly due to the relevant aluminum oxide content^32–40^. The Portland cement chosen had a high initial strength (clinker and calcium sulfate content ≥ 95%), which contains the lowest content of clinker additions and has a higher hydration rate in the first days of curing, aiming to represent a Portland cement with minor additions, similar to CEM I of the European norm EN 197-1^41^. This enabled a better differentiation between the filler and the pozzolanic effect of SCM. SF is available in the Brazilian market and was obtained from the manufacturing process of silicon metal or iron silicon. QP was obtained in laboratory by grinding normalized quartz sand in a planetary mill. For milling, zirconia vessels and spheres were used at a speed of 300 revolutions per minute. After milling, quartz was sieved. The material sieved in a 25-µm mesh sieve and retained in the 20-µm mesh was selected (quartz powder). For the fine aggregate, normalized natural quartz sand was used in the 0.15 mm, 0.30 mm, 0.60 mm and 1.15 mm particle size fractions^42^. The chemical composition of the materials is summarized in Table 1 and the specific surfaces of the cement (Blaine permeameter) and of SCM (adsorption analysis of N_2_ by the BET method) are shown in Table 2.

**Table 1:** Chemical characterization of materials.

Materials	LOI	SiO_2_	CaO	Al_2_O_3_	Fe_2_O_3_	MgO	SO_3_	Na_2_O	K_2_O	Other
	% in mass									
PC	4.3	14.9	58.1	7.0	3.2	3.0	5.3	1.2	0.9	6.4
SF	2.7	91.0	1.0	0.7	0.2	1.7	0.3	0.7	1.0	3.5
QP	0.2	98.0	–	–	–	–	–	–	–	2.0

*LOI* loss on ignition.

**Table 2:** Specific surface of materials obtained by the Blaine (Portland cement) and BET (silica fume and quartz powder) methods.

Materials	PC	SF	QP
Specific surface area (m^2^/g)	0.5	16.3	0.2

The SF showed results of a specific surface area much higher than the PC and the QP^43–45^, it seems that the particle size of the SF is relatively smaller than the PC and the QP. This statement is confirmed; however, there are difficulties in determining the particle size of SF^46–48^. The smaller mean diameter of the SF provides a more reactive material in cement mixtures, presenting a more significant pozzolanic activity due to its smaller particle size and greater specific surface area^8,49,50^. The average diameter and the 90% diameter of Portland cement were 17.21 and 35.57 µm, respectively. The average diameter and the 90% diameter of the quartz powder were 9.47 and 21.49 µm, respectively.

**Figure 1:**
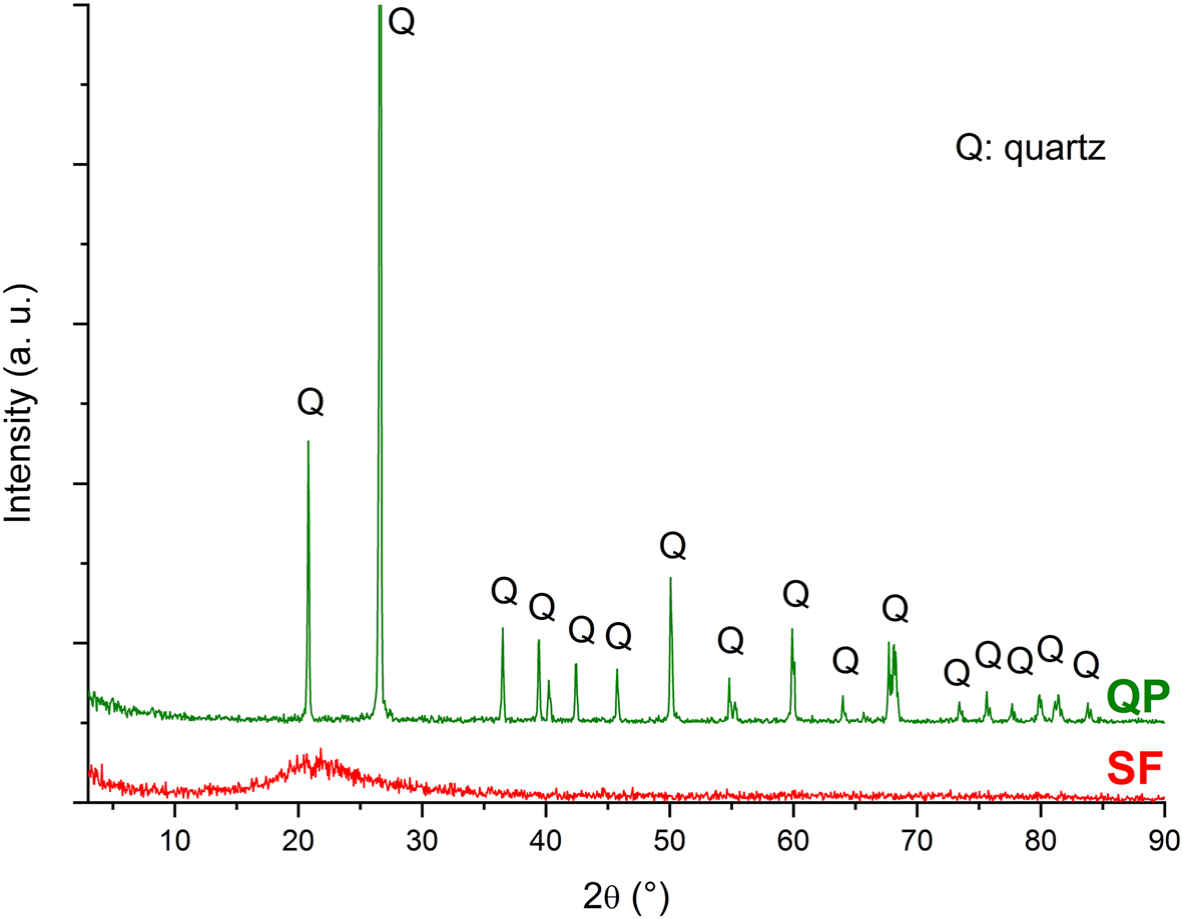
XRD patterns of quartz powder and silica fume.

The X-ray diffraction patterns of SF and QP are shown in Fig. 1. SF has a diffuse dome at 13°–32° 2θ, and is almost entirely 100% amorphous^51,52^. Quartz powder has a high crystallinity (98% by Rietveld refinement, in mass), i.e., the milling process has little influence on the long-range ordering of quartz despite the high fineness of the particles obtained^29,52^.

Thus, silica fume has a specific surface and an amorphous silica content, according to XRD, higher than that of quartz powder, which directly affects the chemical and physical reactivity of these materials. The pozzolanic activity indexes of SF and QP expressed as Ca(OH)_2_ content fixed by the Modified Chapelle Assay^53^, are presented in Table 3. The minimum content for a material to have a pozzolanic activity is 330 mg of lime fixed by 1 g of pozzolan^54,55^, which is equivalent to 436 mg of Ca(OH)_2_ fixed by 1 g of pozzolan. Therefore, silica fume acts as a pozzolanic material, while QP has only a filler effect.

**Table 3:** Modified Chapelle Pozzolanic activity Index.

Samples	Fixed Ca(OH)_2_ content (mg/g)
SF	926
QP	269

### Methods

The mortars and pastes cement were subjected to fresh state tests (flow table consistency, semi-adiabatic calorimetry, and isothermal calorimetry). In the hardened state, the microstructure (using X-ray diffraction, thermogravimetry and its derivative, and scanning electron microscopy), mechanical properties (compressive and tensile strength and modulus of elasticity) and durability parameters (volumetric electrical resistance, water absorption by immersion, accelerated carbonation, alkali-aggregate reactions and Le Chatelier heat expansion) were evaluated.

#### Manufacturing cementitious pastes and composites

Pastes were made with 0.55 water/(cement + SCM). In reference paste (RP), the binder used was Portland cement without SCM. In the other samples, 20% of the mass of the cement was replaced for silica fume (Silica fume paste—SFP) or quartz powder (Quartz powder paste—QPP) for the semi-adiabatic calorimetry and Le Chatelier cold expandability tests.

Mortars (composite call) were made at a ratio of one part cement + SCM, three parts of fine aggregate and 0.55 water/(cement + SCM). In reference composite (RC), the binder used was Portland cement without SCM. In the other samples, 20% of mass of the cement was replaced for silica fume (Silica fume composite—SFC) or quartz powder (Quartz powder composite—QPC).

All composites were prepared using a mechanical mixer. The mixing was carried out according to the Brazilian standard NBR7215^56^. For each mortar type, nine 100 mm × 200 mm cylindrical specimens were made for the modulus of elasticity, volumetric electrical resistance and compressive strength tests. Three other 100 mm × 200 mm cylindrical specimens from each sample were prepared for the accelerated carbonation assay. Three prismatic specimens 40 mm × 40 mm × 160 mm and three cylindrical 50 mm × 100 mm specimens were cast for the flexural strength and immersion water absorption tests, respectively. All specimens were demolded after 24 h and subjected to submerged curing in water until completing the required curing age for each assay. To assess the accelerated expandability due to alkali-aggregate reactions, mortar prisms were prepared using soda-lime glass residue as fine aggregate^57^.

#### Analysis of hydration heat, expandability and consistency

In the cementitious pastes, the heat of hydration in the first hours was analyzed by semi-adiabatic calorimetry using the Adiacal Sn 1272434 Gracial equipment and using a high precision isothermal calorimeter (I-Cal 2000 HPC equipment, Calmetrix brand) to measure the flow heat the cement paste. Also, in pastes, the occurrence of volumetric expansions in the expandability test was studied using Le Chatelier needles^58^. The consistency of cementitious composites was evaluated in the flow table test according to ASTM^59^.

#### Microstructure analysis

The phases in the hardened composites were identified by X-ray diffraction (XRD). Samples after 28 days of curing were macerated in porcelain mortar and pistil and sieved. The passing material in sieve no. 200 (75 µm) was used. The XRD patterns were obtained by Empyrean Panalytical diffractometer using CuKα radiation (40 mA and 40 kV), scanning between 3.03°–89.97° and 0.06° per second.

The calcium hydroxide content present in each type of compound was evaluated by thermogravimetry thermal analysis (TGA) and its first derivative (DTG). A TGA-51 Thermogravimetric Analyzer (Shimadzu) was used at a heating rate of 10 °C/min up to 800 °C in a nitrogen gas atmosphere (N_2_). The analyzed pastes were produced in a controlled, climatized laboratory isolated in plastic film for curing for 28 days. Then, they were crushed, immersed in propanone for hydration interruption, washed with ether and kept in vacuum desiccator in the presence of silica gel according to procedures reported in the literature^60^. The calculation of calcium hydroxide content in the samples considered the carbonation effect according to Rupasinghe et al.^61^. Based on the calcium hydroxide content in the RC, SFC and QPC composites, the lime content determined by the pozzolanic activity of the silica fume and quartz powder was determined^61,62^.

Hydrate formation and microstructure of composites were observed by scanning electron microscopy (SEM). The specimens fractured into pieces were coated with carbon and submitted to SEM analysis using the microscope FEI Quanta 200 FEG under 5 kV of tension.

#### Elastic modulus, compressive and flexural strength

The strength tests (compressive and flexural strength) were performed in an INSTRON 5582 universal machine under a loading rate of 0.25 ± 0.05 MPa/s^56,63^. Compressive strength was measured after 1, 3 and 300 days of curing, and flexural tensile strength was evaluated at 28 days. At each time, three specimens were used per composite type. The elasticity modulus, at 28 and 300 days of curing, was obtained by forced longitudinal resonant frequency^64^ using an Erudite MKII^65^ under a tension of 0.50 V and a frequency range between 7000 and 12,000 Hz.

#### Water absorption by immersion

For the water absorption by immersion test, specimens with 28 days of curing were previously oven-dried at a temperature of 105 ± 5 °C. Specimen mass was measured after three, six and 24 h of drying to ensure that the mass variation was less than 0.5%. Then, the specimens were desiccated for dry mass measurement. Next, the specimens were submerged in water for 28 days at a temperature of 23 ± 2 °C to obtain the saturated mass and the water absorption content by immersion^66^.

#### Volumetric electrical resistance

Volumetric electrical resistance was measured by the two-point method^67,68^ at 300 days of curing. For the test, the specimens were removed from the submerged curing and immediately allocated between two 100 × 100 mm square section copper plates. Wet steel wool was used as a contact between the specimen base and the copper plates. The plates are connected to a known resistor to form a series circuit between the specimen and the resistor. This circuit is subjected to a voltage of 8 V with a frequency of 40 Hz.

#### Accelerated carbonation and expansion due to alkali-aggregate reactions

The accelerated carbonation test was performed using a Thermo Fisher Scientific 3000T RCO-5-VBC chamber. Based on the recommendations of Rilem^69^, specimens with 28 days of curing were placed in the chamber and exposed to an atmosphere with 5% CO_2_ concentration, 48% humidity, and 27.5% ± 2.0 °C for 28 days. Samples were sawed parallel to the bases, and the carbonation depth was determined using phenolphthalein alcohol solution spray and an analog caliper.

The occurrence of possible alkali-aggregate reactions was verified based on ASTM C 1260^57^ using a Solotest thermal bath, greenhouse and dial indicator system. The composites were immersed into 1 mol/L aqueous NaOH solution, and the expansion was measured daily between one and 30 days of curing.

## Results and discussion

### Hydration heat, expandability and consistency

The RP, SFP and QPP pastes did not obtain measurable expansions in the Le Chatelier expandability test. Similar behavior has been observed in studies on the expandability of blast furnace slag cement^70^, soda-lime glass waste^71^, and marble residues^72^. To start the discussion on calorimetry, it is important to note that experimental and precision differences observed between semi-adiabatic and isothermal calorimetry methods are not directly comparable and sometimes with significant differences^73,74^. The results of the semi-adiabatic paste calorimetry test (Table 4) allow identifying the end of the induction period and the maximum temperature of the exothermic peak associated with the acceleration of clinker hydration^75,76^. The partial replacement of cement for silica fume (SFP) and quartz powder (QPP) reduced the maximum temperature reached to RP, indicating that these materials can lower the heat of hydration^30,77,78^. In the case of a filler, this temperature reduction can be attributed to the dilution of clinker^8,21^. The partial replacement of cement for pozzolanic materials can also reduce the heat of hydration as these reactions occur at slower rates^4,76,78,79^.

**Table 4:** Start/end times and maximum temperature of the exothermic peak in semi-adiabatic calorimetry curves.

Paste	Peak formation start time (min)	Maximum peak temperature reaching time (min)	Maximum peak temperature (°C)	Peak acceleration rate (°C/min)
RP	126	480	88.1	0.60
SFP	108	453	75.0	0.36
QPP	148	516	71.2	0.32

The RP paste presented the highest hydration acceleration rate at the peak (0.60 °C/min). This is because the RP paste has a higher cement content and the kinetics of pozzolanic reactions in SP paste is slow in the first hours of curing. As for the peak formation onset times, silica fume anticipated the occurrence of the exothermic peak, while quartz powder delayed the cement hydration to the RP mixture. Thus, in the first hours of hydration, the heterogeneous nucleation provided by silica fume appears to be more pronounced than that of quartz powder^30^. Silica fume has a much larger specific surface (~ 90 times) (Table 2), i.e., it has a larger surface area available for nucleation of hydrated phases^19^. Considering that the use of silica fume anticipated the formation of the peak but reduced its intensity, it can be inferred that its effect during the first hours of curing consists mainly in physically stimulating cement hydration with little influence of pozzolanic activity^19,23,80^. The small specific surface of quartz powder, in turn, did not favor nucleation, which delayed the hydration of the QP paste.

**Figure 2:**
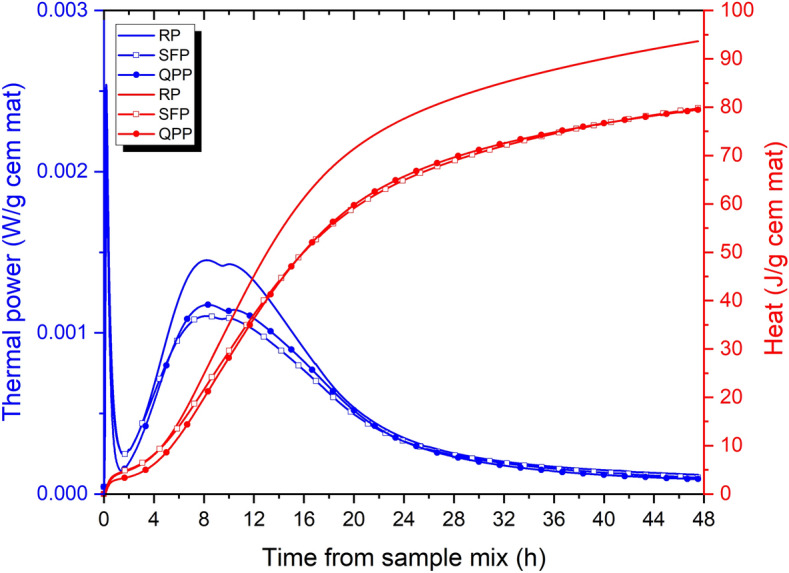
Thermal power and accumulated heat curves of RP, SFP and QPP (Isothermal).

The thermal power and accumulated heat normalized per gram of cementitious material are shown in Fig. 2. The thermal power curve has a pre-induction period between 1 and 2 h. It is noticed that between 2 and 48 h, the behavior of SFP and QPP are similar. Both have lower thermal power than RP. After this the maximum thermal power value of the three curves, there is the next stage, the deceleration. The substitution of part of the RC by SF and QP promotes the prolongation of the induction phase, decreases the exothermic peaks and reduces the reaction rate. The first peak (~ 8hs) of thermal power that is responsible for the hydration of the primary silicate^81,82^, did not have its occurrence time significantly altered by the substitution of RC by SF and QP. This indicates that the SF and the QP did not accelerate the hydration processes of the RC, but rather decrease the heat release as a function of time, corroborating the results of the peak acceleration rate (°C/min) of the adiabatic calorimetry. This first peak is also attributed to the dissolution of C_3_S, this decrease in its height is due to the smaller amount of RC^83^. The same happens for the second peak (~ 11hs) generated by the hydration of the aluminates^81^. The hydration heat of the RP was determined to be 93.562 J/g of cementitious material. SFP and QPP showed heat lower than RP, 79.758 and 79.488 J/g of cementitious material, respectively.

**Figure 3:**
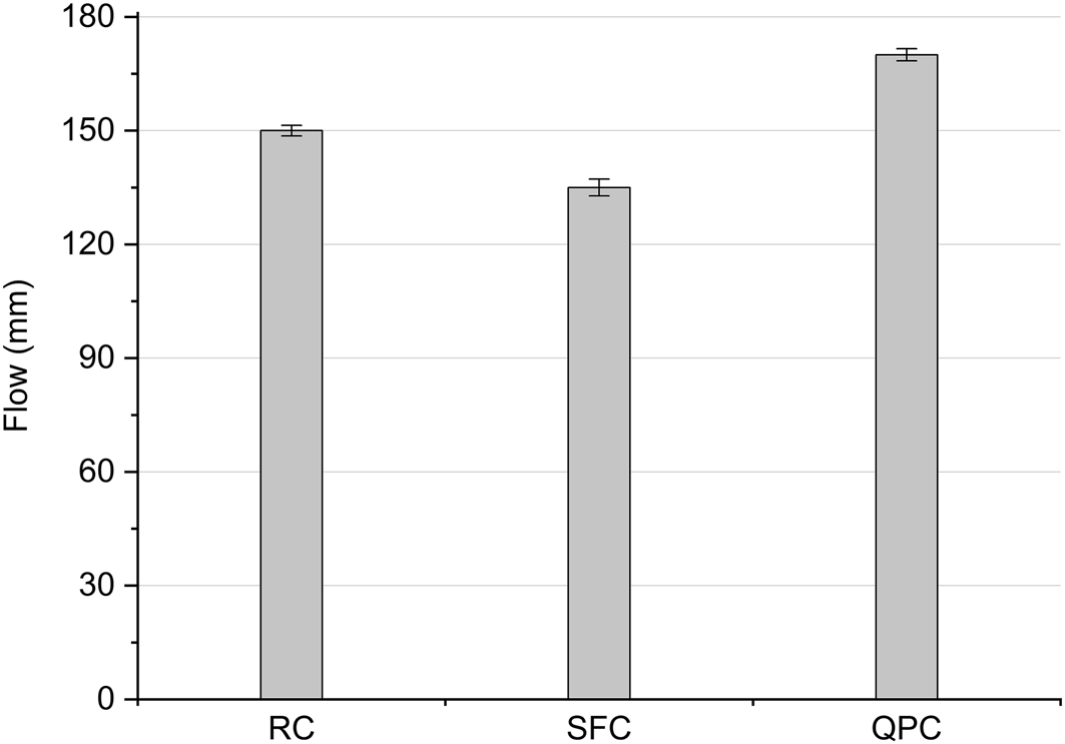
Flow table of RC, SFC and QPC composites.

The flow for cementitious composites is shown in Fig. 3. The composite SFC had the lowest flow, while the composite QPC had a greater flow than the RC. The great fineness of silica fume contributed to the increased demand for water in the mixture, which has reduced the scattering of the composite SFC^12,84^. On the other hand, as for the QPC composite, replacing cement for a low specific surface material increases the flow of composites in the fresh state. In fact, other studies indicate that the partial replacement of cement for chemically inert materials (filler) tends to reduce the water demand in the mixture and increase the flow or workability^72,85^.

### Analysis of hydration phases and microstructure

**Figure 4:**
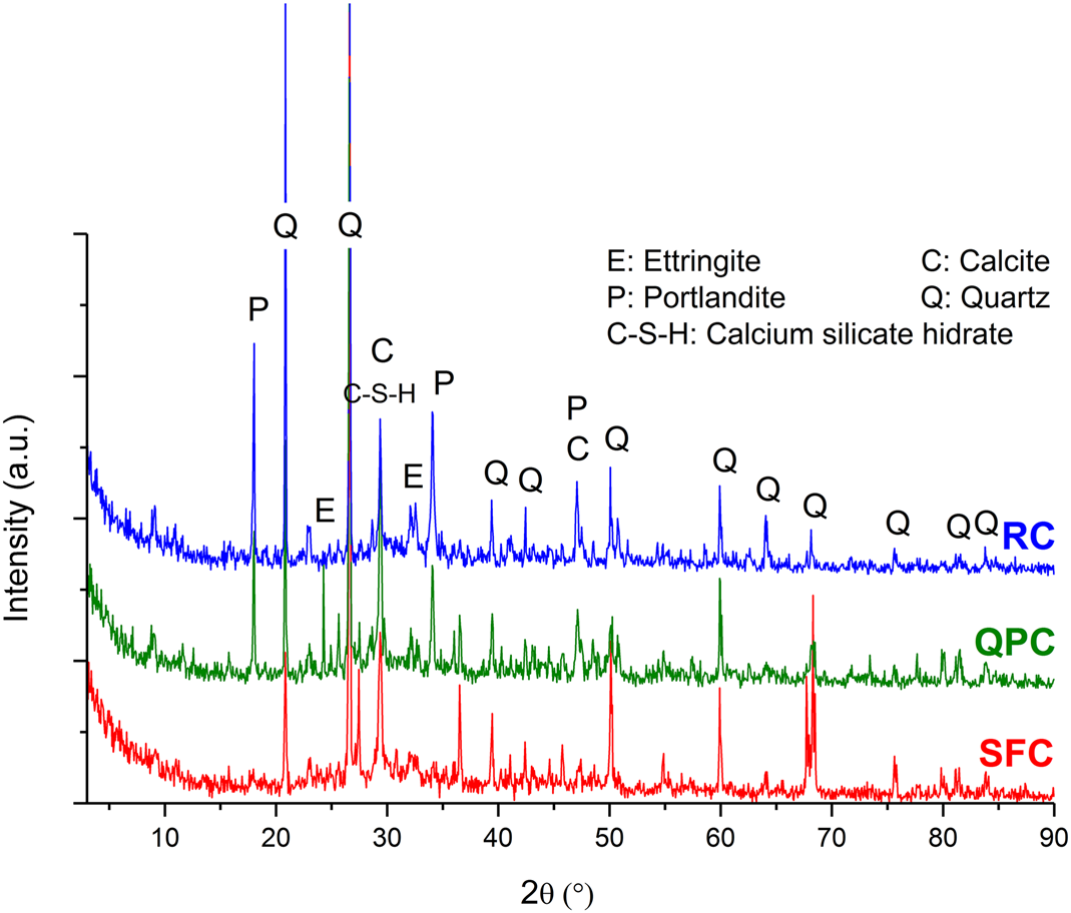
XRD traces of the RC, SFC and QPC.

The XRD patterns of the composites at 28 days of curing are shown in Fig. 4. All three composites have similar patterns. Quartz peaks are associated with fine aggregate, and calcite peaks can be attributed to sample carbonation. The presence of hydrated ettringite and calcium silicate originating from the hydration of Portland cement was identified in all samples^86–88^. However, portlandite was detected only in the RC and QPC, indicating that, after 28 days of curing, the pozzolanic activity of silica fume contributed to increasing the density of C–S–H present in the cementitious composite microstructure^12^. The composite QPC, in turn, had a XRD pattern very similar to that of the RC, indicating that there was a low reactivity of quartz powder to hydrated cement^30,80^.

**Figure 5:**
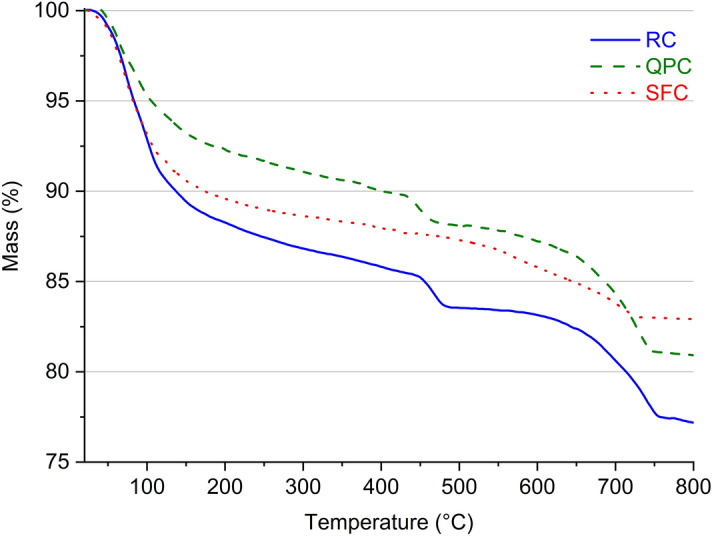
TGA curves of the RC, SFC and QPC.

The thermogravimetry (TGA) of composites at 28 days of curing are presented in Fig. 5. The first two peaks found in DTG curves (62–73 °C and 89–102 °C) are due to the decomposition of ettringite and the C–S–H, respectively^24,62,89^. The loss of free water and physically combined water is also concentrated within these temperature ranges^78,89^, which contributed to both peaks occurring before 100 °C, being associated with C–S–H and AFm^90^. The third and fourth peaks (423–468 °C and 719-738ºC) are mainly due to the decomposition of calcium hydroxide and calcium carbonate, respectively^61,90^. The SFC decomposes calcium hydroxide and calcium carbonate noticeably lower than RC and QPC, which was due to the high pozzolanic activity of silica fume^12,91–93^.

**Figure 6:**
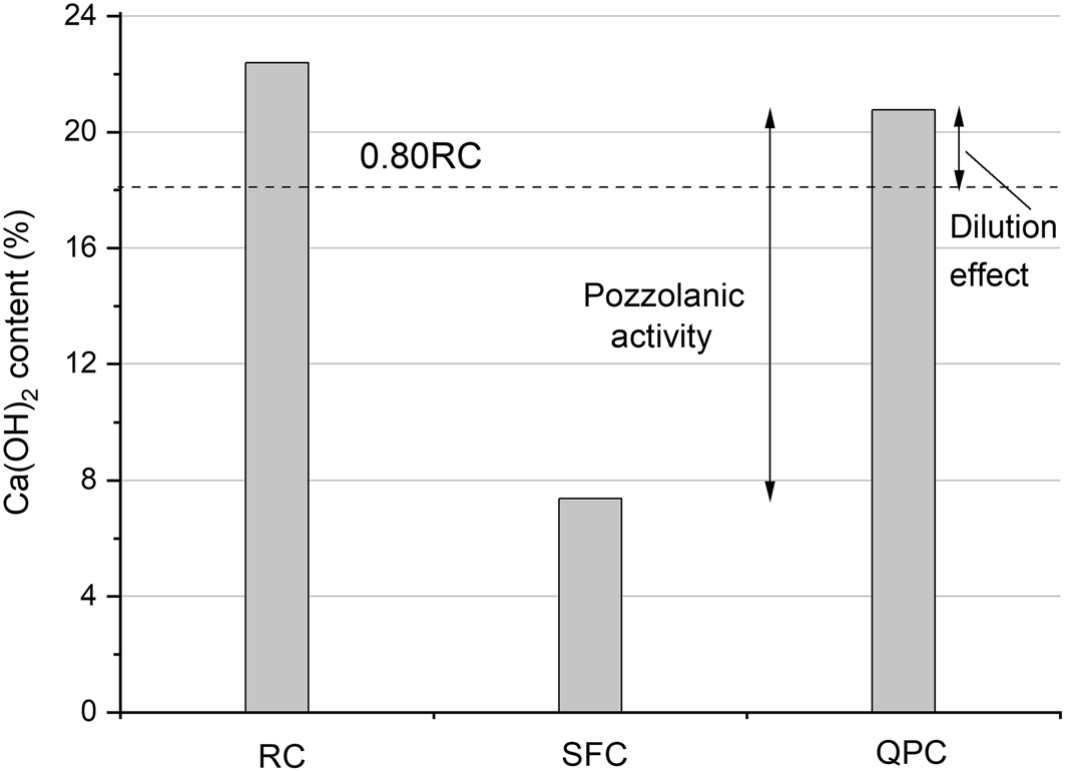
Ca(OH)_2_ contents of the RC, SFC and QPC.

Calcium hydroxide contents in the RC, SFC and QPC composites at 28 days of curing are shown in Fig. 6. Considering that the SFC and QPC binders have 80% of cement in mass, the Ca(OH)_2_ contents in these composites were compared to the 0.80RC (Ca(OH)_2_ content of the sample RC multiplied by 0.80). The use of quartz powder increased the Ca(OH)_2_ content by 16% over the 0.80RC value, which can be attributed to the filler (nucleation) effect or the extra space available for the formation of hydrates due to clinker dilution^21,24,80^. Since semi-adiabatic calorimetry analysis indicated that the nucleation effect of quartz powder particles was insignificant (item 3.1), this increase in Ca(OH)_2_ content is due to the dilution effect of the cement.

The SFC, in turn, had a Ca(OH)_2_ content 59% lower than the 0.80RC due to Ca(OH)_2_ consumption by the pozzolanic reaction^12,24,61,91^. Considering that silica fume particles also act on clinker dilution and can physically accelerate cement hydration (item 3.1), the Ca(OH)_2_ content of SFC was also compared to that of QPC. In this case, silica fume consumed almost 65% of the Ca(OH)_2_ produced by cement hydration.

**Figure 7:**
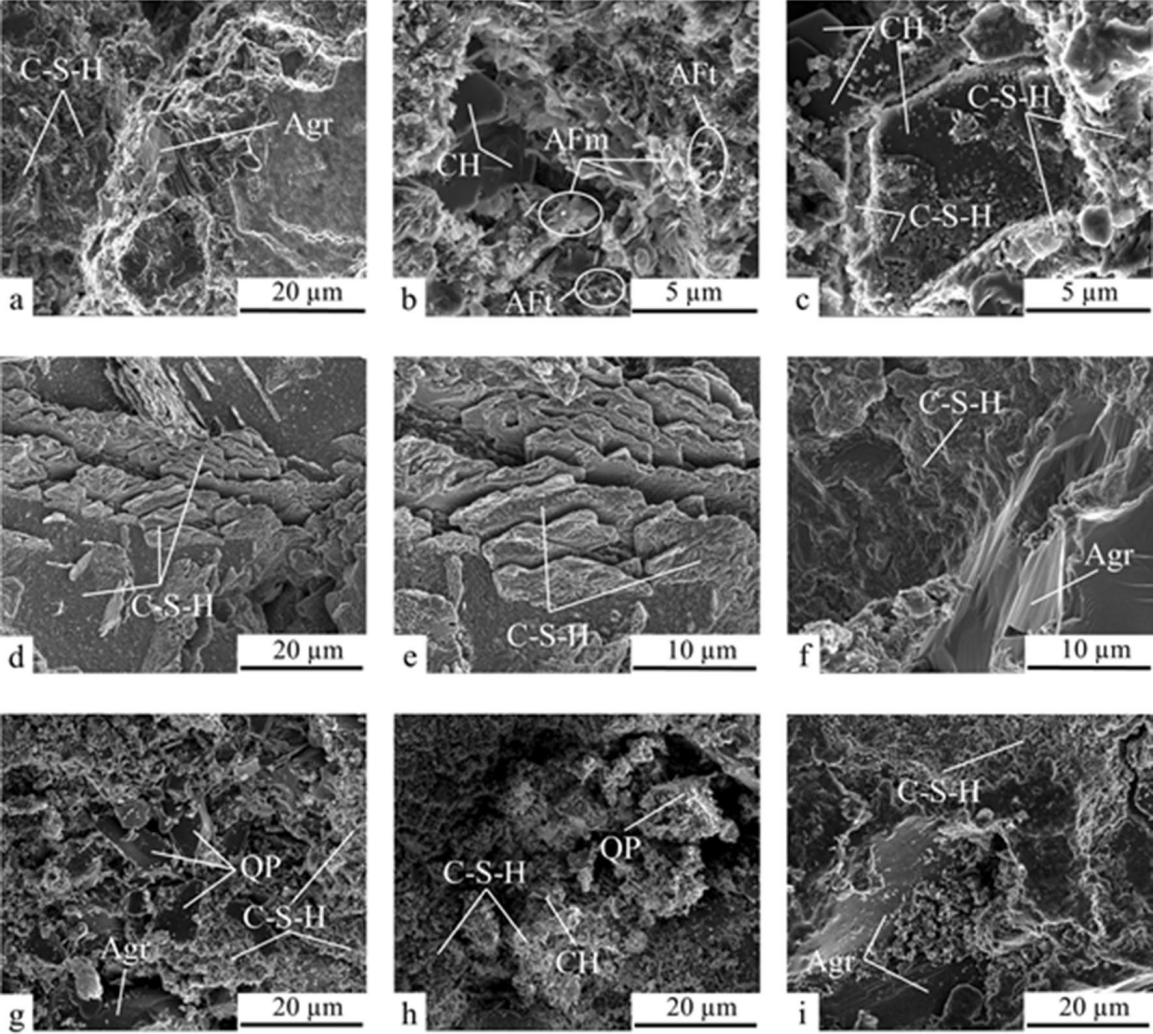
SEM (**a**–**c**) analysis of RC, (**d**–**f**) SFC and (**g**–**i**) QPC. Note: AFm—monosulfate; AFt—ettringite; Agr—fine aggregate; CH—calcium hydroxide; C–S–H—calcium silicate hydrate; and QP—quartz powder.

The composite images by SEM are presented in Fig. 7. The images 7a-c show that the RC microstructure is mainly composed of calcium silicate hydrate (C–S–H) that surround fine aggregate particles (Agr) and other hydrated compounds such as calcium hydroxide lamellar particles (CH), monosulfate (AFm) and needle-shaped particles of ettringite (AFt)^12,94–97^. In addition, C–S–H on the surface of calcium hydroxide crystals appear to have been detected (Fig. 7c), indicating that the microstructure contains a C–S–H with a higher Ca/Si ratio and a higher calcium hydroxide concentration^98^, which could slow the advancement of the carbonation front in the composite RC (Fig. 10).

The images 7d-f indicate that pozzolanic activity reduced pore size and made the SFC microstructure denser and more compact^12,91,99^, whereas crystals of Ca(OH)_2_ were not detected^96^. This corroborates the high Ca(OH)_2_ consumption determined in the TGA analysis of the SFC. Moreover, as shown in Fig. 7f, the paste-aggregate interface can also be densified due to secondary C–S–H formation by pozzolanic reactions^99^. This indicates that the pozzolanic activity could cause improvement in mechanical properties, reduction of permeability, and an increase in electrical resistance of composites, which will be discussed in the following items.

The QPC, on the other hand, presented a less dense and more porous microstructure (Fig. 7g–i). The images Fig. 7g and Fig. 7h suggest that quartz powder microparticles hardly acted as nucleation point, and interacted poorly with hydrated cement^30^. The image Fig. 7i shows that the paste-aggregate interface in QPC is weaker than in SFC. Thus, although cement dilution stimulated clinker hydration due to the extra space for hydrate growth, as suggested by TGA analysis (Fig. 6), the increase in porosity caused by cement dilution and low powder reactivity of quartz powder with hydrated cement were preponderant for the porous microstructure of the hardened QPC composite. Thus, the SCM reactivity (in terms of pozzolanic activity and filler effect on new nucleation points) plays an essential role in the development of the microstructure of cementitious composites.

### Strength and elastic modulus

The compressive strength values of composites after one, three and 300 days of curing, flexural strength at 28 days, and elasticity modulus at 28 and 300 days are presented in Table 5. All samples showed a monotonic growth of compressive strength over time. The RC obtained strength gains of 39% between the first and the third day of curing and 33% in the interval between three and 300 days of curing. The strength of QPC increased by 82% (66%) between the first and the third day of cure and by 126% (41%) between three and 300 days of curing.

**Table 5:** Mechanical properties of composites.

	Composites		
	RC	SFC	QPC
**Compressive strength (MPa)**			
1 day	29.1 [2.9]	14.1 [1.8]	21.2 [1.8]
3 days	40.5 [3.3]	25.7 [2.7]	21.0 [3.2]
300 days	53.8 [4.2]	58.1 [4.7]	49.6 [3.1]
**Flexural strength (MPa)**			
28 days	7.4 [0.4]	7.8 [0.4]	6.8 [0.7]
**Elasticity modulus (MPa)**			
28 days	36.0 [1.2]	28.5 [0.9]	31.6 [1.8]
300 days	37.2 [1.3]	35.0 [2.7]	33.6 [2.1]

An important fact to report is that the compressive strength shown by SFC and QPC are significantly lower than RC at 1 and 3 days. This is reinforced by the results of semi-diabetic and isothermal calorimetry, where higher peak temperature (°C), peak acceleration rate (°C/min), thermal power and heat normalized per gram of cementitious material are verified. The higher compressive strength value of the composite RC in the early times was due to the performance of the high initial strength cement. In addition, the low reactivity of silica fume in the first hours of curing (Fig. 2) contributed to the lower compressive strength of the SFC up to three days of curing. After this period, the increase in strength of the SFC was more significant due to the pozzolanic activity of the silica fume^100–102^. Still, the strength of SFC and RC were statistically equal at 300 days. The QPC always had an equivalent strength compared to the reference composite. Similar behavior was observed in mortars containing 15% of marble residue^72^. Antoni et al.^80^ found that mortars containing a mixture of metakaolin and lime filler had higher strength than mortars containing the same quartz powder contents. The difference between the strength of RC and QPC is due to the microstructure porosity. While the pozzolanic activity densified the microstructure in the SFC, the low reactivity of quartz was not enough to compensate for the porosity increase caused by clinker dilution (Fig. 7).

The composites RC, SFC and QPC presented similar flexural strength values considering the scattering of measurements. This behavior is similar to that observed in other studies^12,72,103^, in which the use of filler or pozzolanic materials had little influence on the tensile strength of cementitious compounds.

The modulus of elasticity values of the three samples is within the same measurement range at 300 days of curing. Considering only mean values, the composite SFC presented a higher average compressive strength but a lower average modulus of elasticity to the composite RC. Similar behavior was reported by Giner et al^104^ in a study of static and dynamic mechanical properties of silica fume concretes. Youm et al.^105^ found that silica fume-containing cement can increase the strength of concretes but without significant static modulus gains. Thus, cement with silica fume and quartz powder had little influence on the elasticity modulus.

### Water absorption by immersion

**Figure 8:**
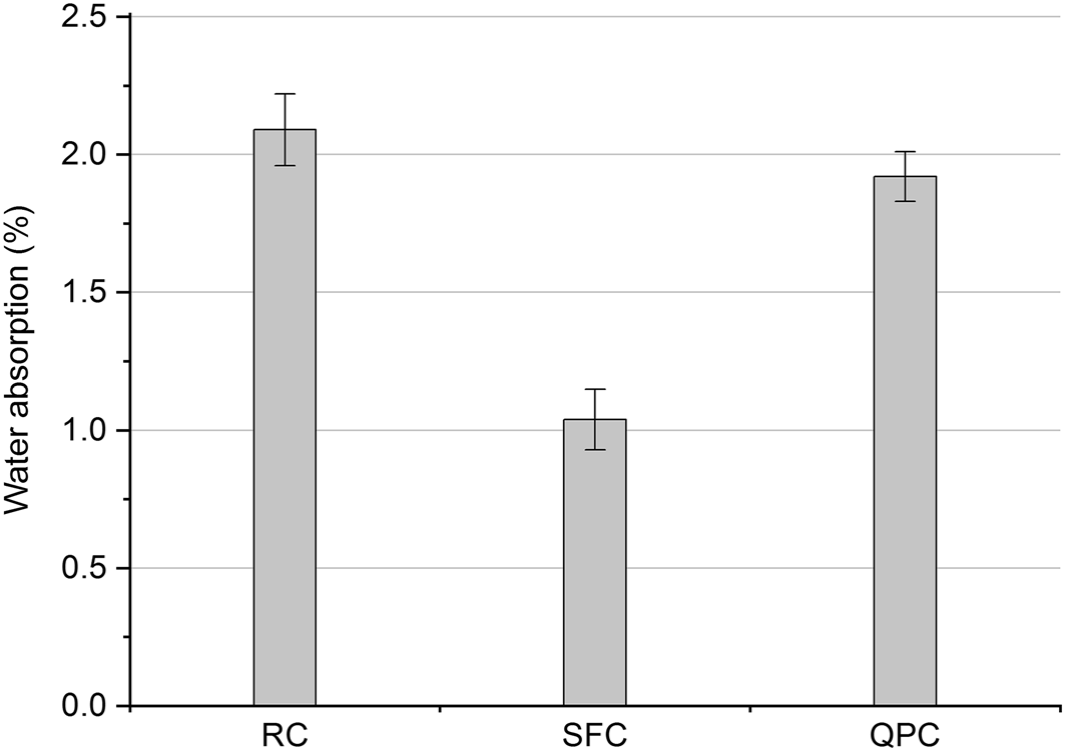
Water absorption by immersion of the RC, SFC and QPC at 28 days of curing.

The composites' water absorption by immersion after 28 days of curing are shown in Fig. 8. The composites SF and QPC had a reduction in water absorption by 50% and 8%, respectively, compared to the RS composite. This indicates that pozzolanic activity acted more significantly in pore refinement and decreased water absorption to the filler effect^106–109^.

### Volumetric electrical resistance

**Figure 9:**
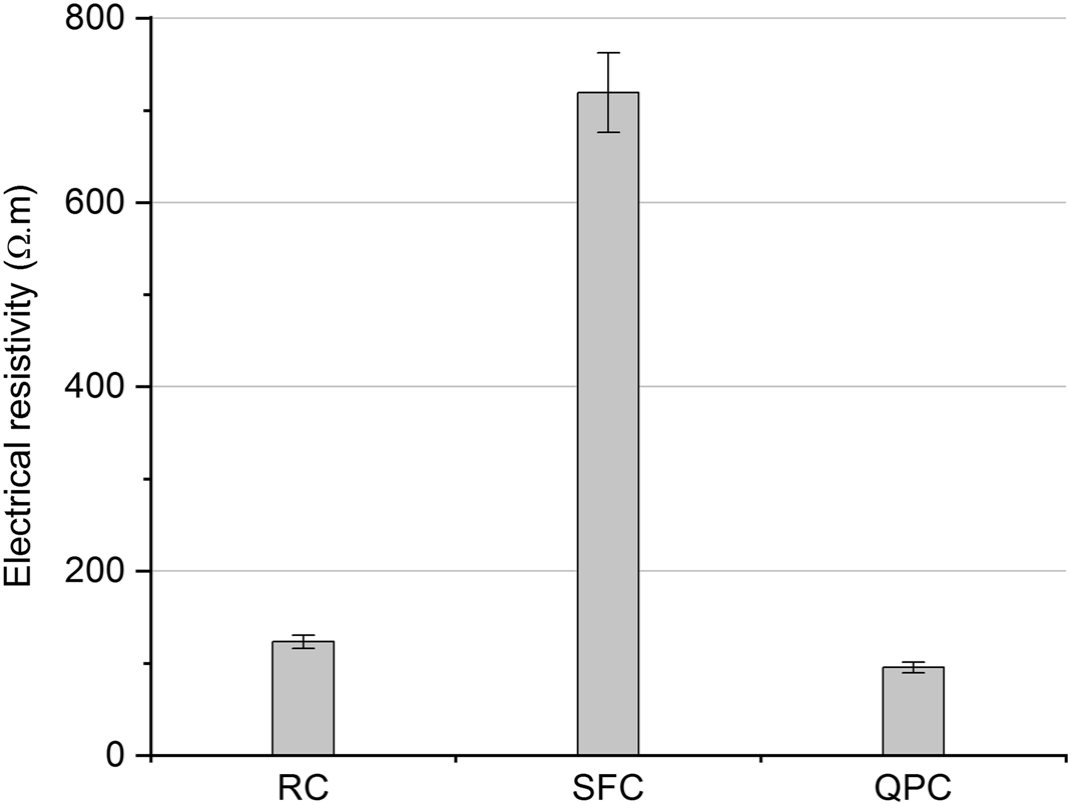
Volumetric electrical resistance of the RC, SFC and QPC at 300 days of curing.

The volumetric electrical resistance of the composites at 300 days are shown in Fig. 9. The electrical resistance of the SFC was seven times higher than that of RC, which can be attributed to pore refinement and reduction of open-pore connectivity due to pozzolanic reactions^25,91,110^. On the other hand, the use of quartz powder reduced electrical resistance by almost 25%. Medeiros-Júnior and Lima^25^ reported that cement containing filler might not contribute to increasing the electrical resistance of the concrete compared to a high initial strength cement. Thus, the pozzolanic activity exerts a more significant influence on both the increase in electrical resistance and the reduction of water absorption (Fig. 8) to the filler effect.

Many studies have correlated the electrical resistance of concrete with the risk of reinforcement corrosion^67,110,111^. According to the classification proposed by AASHTO TP119^112^, concretes with a volumetric electrical resistance within 52–104 Ω m have a moderate chloride ion penetration, while resistance values within 104–208 Ω m and 208–2070 Ω.m indicate a low and a shallow chloride ion penetration, respectively. Considering that the risk of corrosion in steel bar reinforced by the composite is related to chloride penetration, the use of silica fumes is efficient to minimize the risk of corrosion. On the other hand, reinforced concrete containing quartz powder has a considerable risk of reinforcement corrosion. Thus, the pozzolanic activity seems to influence more strongly the durability parameters against corrosion than the filler effect.

### Accelerated carbonation

**Figure 10:**
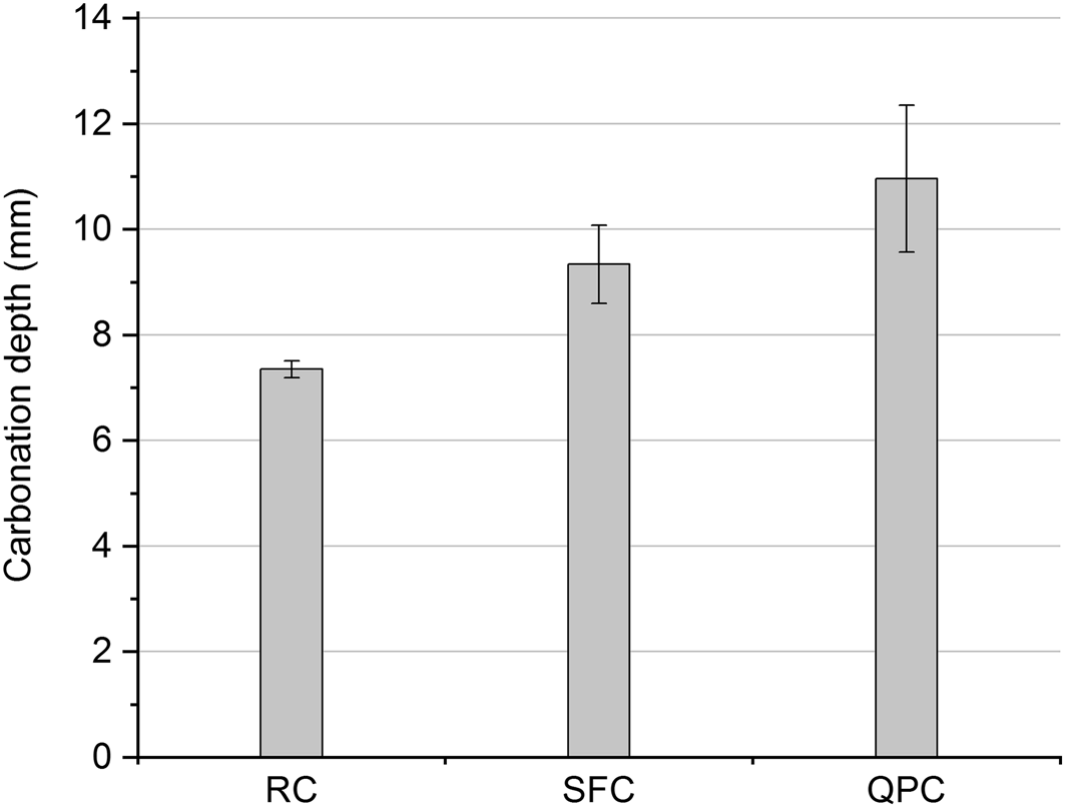
Accelerated carbonation depth of RC, SFC and QPC.

The accelerated carbonation depth values of the composites studied are shown in Fig. 10. The use of quartz powder implied a greater carbonation depth, which can be attributed, first of all, to cement dilution^85^. As previously indicated, the low reactivity of quartz powder was not enough to reverse the increase in microstructure porosity caused by cement dilution (Fig. 7). This fact contributed to the lower values of electrical resistance and water absorption in the QPC composite (Figs. 8 and 9) and favored the penetration and diffusion of CO_2_ in the cementitious matrix and the advance of the carbonation front.

The use of silica fume, in turn, proved to be efficient in terms of increasing electrical resistance and reducing water absorption. However, it increased carbonation depth in relation to the RC composite. This behavior is due to two distinct effects of pozzolanic reactions on cementitious composites. First, as previously indicated, pozzolans can physically and chemically interact with hydrated cement, provide pore refinement and increase the density of the cementitious matrix microstructure (Fig. 7)^12,80,113,114^. This effect hinders the diffusion of carbon dioxide (CO_2_) into the remaining pore solution^26^ and explains the improvement in electrical resistance and water absorption in the RC composite. However, secondly, the pozzolanic reactions consume part of alkaline reserves and reduce the pH in the pore solution of the cementitious matrix, which accelerates the reaction of CO_2_ with the alkali in the pores and, consequently, favors the advance of the carbonation front^9,115^. The TGA and DTG curves of the SF composite (Fig. 5) indicate a low Ca(OH)_2_ content remaining in the microstructure. Thus, the advancement of the carbonation front in the composites seems to be more sensitive to pore alkalinity reduction than to pore refinement and microstructure densification. This explains the increased carbonation depth of the RC composite despite the significant increase in volumetric electrical resistance and reduced water absorption by immersion.

### Expansions due to alkali-aggregate reactions

**Figure 11:**
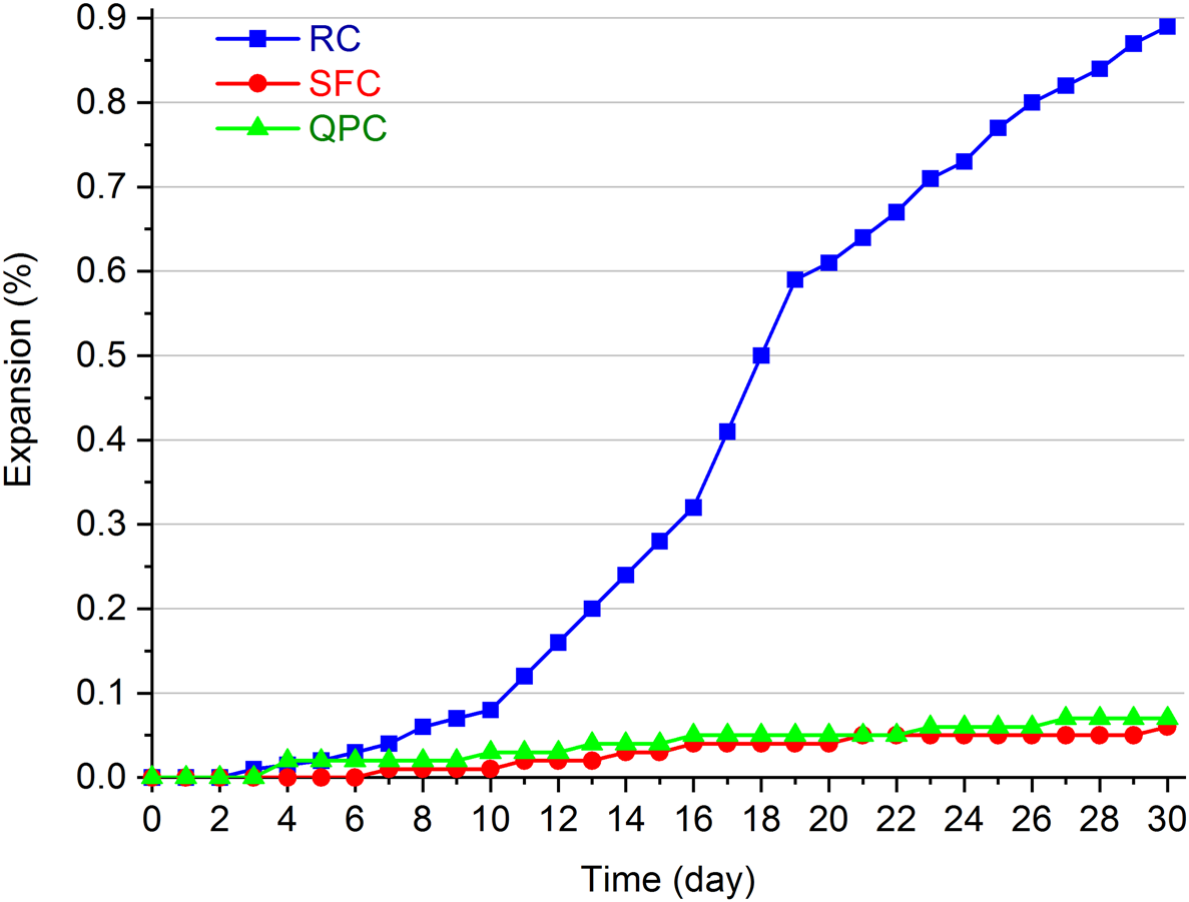
Expansion by alkali-aggregate reactions using the mortar-bar method in the RC, SFC and QPC.

The risk analysis of alkali-aggregate reactions in the mortar-bar method are shown in Fig. 11. According to ASTM C-1260^57^, an expansion of 0.10–0.20% on the 16th test day could indicate the occurrence of a deleterious reaction. The composites RC and QPC showed little significant expansion until 30 days of curing, indicating a low probability of alkali-aggregate reactions. This result is in agreement with other studies in which the use of silica fume and allowed the reduction of expansion caused by alkali-aggregate reactions^116,117^.

The SFC has less expansion than the composite RC. The pozzolanic activity of silica fume contributes to the formation of hydrous calcium silicates with a denser microstructure and a lower Ca/Si ratio, which favors the absorption of alkalis by C–S–H. This decreases the concentration of alkali in the pores and thus restrict the occurrence of alkali-aggregate reactions^118,119^. In the composite QPC, the cement dilution generated a porous microstructure (Fig. 7) with a void volume that may be sufficient for the formation of alkali-aggregate reaction expansive products without significant expansions in the specimen. These hypotheses agree with the accelerated carbonation test (Fig. 10), in which pore alkalinity reduction and cement dilution, respectively, seem to be preponderant for the advancement of the carbonation front in the RC and QPC.

## Conclusion

When analyzing semi adiabatic calorimetry, the partial replacement of cement for silica fume and quartz powder reduces peak temperatures to the reference paste, indicating that these materials reduce the heat of hydration. Silica fume anticipates the occurrence of exothermic peaks, while quartz powder delays cement hydration to the reference mixture. This indicates that in the first hours of curing, silica fume only interacts with the hydrated cement physically. Also, the filler effect of silica smoke on new nucleation points is more intense than that of quartz powder due to the high specific surface difference between these materials. However, when the isothermal calorimetry is analyzed, it is possible to perceive very clearly the reduction of the thermal energy released in the hydration process, with no acceleration or deceleration in the hydration reactions being noticeable.

The silica fume composite shows less scattering due to its high specific surface, which contributes to the increase in water demand in the mixture. The quartz powder increases the scattering of composites due to the replacement of cement for smaller specific surface material.

According to the XRD and TGA analyses, the quartz powder composite has the same phases as the reference composite, indicating that there is a low reactivity of quartz powder with the hydrated cement. However, in the silica fume composite, the absence of portlandite observed in XRD and the low Ca(OH)_2_ content determined in the thermal analysis confirm the occurrence of the pozzolanic reaction.

The composite containing quartz powder shows a porous microstructure, which implied higher water absorption by immersion and low electrical resistance. It is possible that the clinker dilution effect favors cement hydration in this composite, as suggested by the TGA analysis. However, the increase in porosity due to dilution seems to have been predominant in the microstructure. The microstructure with less pores of silica fume compounds indicates that the pozzolanic reaction reduced pore size and connectivity, resulting in reduced permeability, and increased electrical resistance of the composites. However, this densification did not increase the carbonation resistance of these compounds. Both silica fume and quartz powder increase the carbonation depth of the composites. This indicates that the effects of pozzolanic reactions on the reduction in alkali content and pH in the pore solution is more significant in the accelerated carbonation test than the microstructure pore refinement.

The higher microstructure density in the silica fume-containing composite considerably reduces the risk of expansion by the alkali-aggregate reaction. The composite containing quartz powder also obtained less expansion in the accelerated mortar-bar test, indicating that the cement dilution can provide a larger volume available for the formation of expansive products.
